# Cnidaria herd optimized fuzzy C-means clustering enabled deep learning model for lung nodule detection

**DOI:** 10.3389/fphys.2025.1511716

**Published:** 2025-03-18

**Authors:** R. Hari Prasada Rao, Agam Das Goswami

**Affiliations:** School of Electronics Engineering, VIT-AP University, Amaravati, Andhra Pradesh, India

**Keywords:** lung nodule detection, fuzzy c-means clustering, Resnet −101, cnidaria herd optimization, lobe segmentation, deep learning

## Abstract

**Introduction:**

Lung nodule detection is a crucial task for diagnosis and lung cancer prevention. However, it can be extremely difficult to identify tiny nodules in medical images since pulmonary nodules vary greatly in shape, size, and location. Further, the implemented methods have certain limitations including scalability, robustness, data availability, and false detection rate.

**Methods:**

To overcome the limitations in the existing techniques, this research proposes the Cnidaria Herd Optimization (CHO) algorithm-enabled Bi-directional Long Short-Term Memory (CHSTM) model for effective lung nodule detection. Furthermore, statistical and texture descriptors extract the significant features that aid in improving the detection accuracy. In addition, the FC2R segmentation model combines the optimized fuzzy C-means clustering algorithm and the Resnet −101 deep learning approach that effectively improves the performance of the model. Specifically, the CHO algorithm is modelled using the combination of the induced movement strategy of krill with the time control mechanism of the cnidaria to find the optimal solution and improve the CHSTM model’s performance.

**Results:**

According to the experimental findings of a performance comparison between other established methods, the FC2R + CHSTM model achieves 98.09% sensitivity, 97.71% accuracy, and 97.03% specificity for TP 80 utilizing the LUNA-16 dataset. Utilizing the LIDC/IDRI dataset, the proposed approach attained a high accuracy of 97.59%, sensitivity of 96.77%, and specificity of 98.41% with k-fold validation outperforming the other existing techniques.

**Conclusion:**

The proposed FC2R + CHSTM model effectively detects lung nodules with minimum loss and better accuracy.

## 1 Introduction

Lung nodule detection is the initial stage of lung cancer screening in clinical medicine (Sharma et al., 2019) enabling prompt and appropriate treatment that can significantly lower the death rate (Schabath and Cote, 2019; Ji et al., 2023) Medical image analysis has long made use of image processing techniques (Naseer et al., 2023). Physicians use a variety of diagnostic techniques to identify malignant lung nodules early on. In clinical settings, these techniques include needle prick biopsy analysis, Computed Tomography (CT) scan analysis, and morphological assessment (Nasrullah et al., 2019; Chen et al., 2021). The hit ratio for manually detecting lung cancer may be lowered by two major issues. The availability of people and technology comes first since there might not be enough radiological resources to meet demand (Thallam et al., 2020). Secondly, a considerable proportion of false positive cases can be attributed to the initial flaw. Thus, radiologists who interpret the images should have excellent training. As a result, improvements are still needed in the detection and classification precision of current systems (Mahum and AlSalman, 2023). Additionally, the tiny size and variety of shapes of pulmonary nodules in general medical imaging further complicate identification and raise the possibility of an incorrect or missing diagnosis (Ji et al., 2023).

Therefore, systems for computer-aided detection (CAD) are suggested as a means of automatically detecting lung nodules in CT scan images. A common diagnostic imaging technique called CT enables radiologists to look for and assess any worrisome lung nodules on CT scans (Nguyen et al., 2021). Conventional CAD systems typically do false positive reduction using traditional classifiers (Murphy et al., 2009; Sui et al., 2015) and produce nodule candidates using manually constructed nodule features such as morphological features (Jacobs et al., 2014) or intensity thresholding (Akram et al., 2016). Due to the abnormal structure of lung tissues (Tong et al., 2020) these techniques rarely produce adequate results (Nguyen et al., 2021). However, the manual techniques require more time and the ML techniques have limited generalization ability because of limited training samples. One method for diagnosing lung cancer is to employ segmentation techniques (Liu et al., 2021a). Based on the image’s color, and texture, the ROI is chosen as a segmented area. Three common segmentation techniques are thresholding, atlas, and region growth. The retrieved attributes of the segmented area have a substantial impact on the performance of segmentation-based methods. Approaches based on segmentation have shown encouraging outcomes in the identification of lung cancer. These methods must be adjusted to reduce their false ratio, as they are still not applicable to samples that have not yet been observed (Mahum and AlSalman, 2023).

Deep learning (DL) approaches have been used with promising results in the categorization of lung nodules in recent years (Chen et al., 2020; Li et al., 2020; Wang et al., 2019; Sreekumar et al., 2020). However, persistently applying local processes within Convolutional Neural Network (CNN) layers to analyse texture characteristics does not adequately capture the intricate composition and long-range relationships present within a lung nodule (Saihood et al., 2023). Additionally, pooling layers serve as the foundation for attention-based multiple-instance learning techniques. Since the features that are retrieved from both modalities have varying characteristics, these models cannot be used statically and must be fused proportionately. Here the step convolution module (SCM), which adopts a parallel structure design and has receptive fields of different sizes and a residual squeeze and excitation attention module (RSEAM), improves the useful feature gain through space and channel. Moreover, it cannot be used statically instead of that a fusion must done proportionately with the U-Net. The U-Net mainly operates to obtain image features through a series of convolutional and pooling operations. However, the RSEA-Net requires a precoding network to provide the gate signal and the SCM structure is complicated (Liang et al., 2022). A feature pyramid network (FPN) (Lin et al., 2017) is a tool used in some techniques to extract multi-scale information from nodules. Many down-sampled features are restored by the feature pyramid through up-sampling; nevertheless, the up-sampled features’ pixel values and positions differ from the original feature images without down-sampling (Zhang et al., 2022). Without human involvement, DL approaches may effectively extract significant features in the best possible way. In the medical field, these methods can increase the accuracy of disease detection (Naseer et al., 2023).

Existing methods have limitations in scalability, robustness, data availability, and false detection rates. To address these issues, a novel deep-learning model is for accurate lung nodule detection. This research aims to employ the FC2R + CHSTM model to identify lung nodules from CT scans. By combining the benefits of Resnet −101 and FCM, the FC2R segmentation model successfully separates the lung nodules from the healthy regions. Furthermore, from the segmentation output, the ResNet-101 features, statistical features, and texture information are extracted which improves the nodule detection accuracy. The combination of the induced movement strategy of krill with the time control mechanism of the cnidaria improves the CHSTM model’s performance to attain better detection accuracy.

### 1.1 Cnidaria herd optimization enabled the Bi-directional long short-term memory

The CHO algorithm mimics the food-searching behaviour of the cnidaria and the herding characteristics of krill. The combination of the induced movement strategy of krill with the time control mechanism of the cnidaria improves the CHSTM model’s performance to attain better detection accuracy.

### 1.2 Optimized fuzzy C-means clustering algorithm and the Resnet -101 deep learning model

The FC2R method for lung nodule segmentation accurately segments the nodule regions using the FCM with the Resnet −101 model. The proposed optimization is used to compute the coefficient features and the Resnet −101 model generates the flow maps that lead to improving the performance of the model.

### 1.3 Cnidaria herd optimization algorithm enabled BiLSTM model with fuzzy C-means clustering algorithm and the Resnet −101 deep learning model

The combined CHSTM + FC2R approach integrates the advantages of bio-inspired optimization algorithm with the novel segmentation technique which aids the model to detect lung nodules with superior performance. In addition, the CHSTM + FC2R model increases the system reliability minimizes the overfitting issues caused by data imbalance problems, and increases the computational efficiency of the lung nodule detection tasks.

The organization of the manuscript is described as follows: Section 2 covers the limits of the linked works’ literature evaluation. Section 3 discusses the suggested methodology for lung nodule identification along with its methodology flow. Sections 4, 5 explain the research outcomes and conclusion respectively.

## 2 Motivation

To detect effective lung nodules many existing methods are utilized and they have limitations in scalability, robustness, data availability, and false detection rates. To overcome all these issues a novel deep learning is developed for accurate lung nodule detection. This research aims to employ an FC2R + CHSTM model to identify the lung nodule from the CT scans. The FC2R segmentation helps to separate the lung nodule from the healthy region. The CHO algorithm helps to fine-tune the hyperparameters of the model and the combination of the induced movement strategy of krill with the time control mechanism of the cnidaria improves the CHSTM model’s performance to attain better detection accuracy.

### 2.1 Literature review

The existing methods implemented for lung nodule detection with their limitations and advantages are explained in the following section.

Zhang et al. (2022) utilized an attention deep learning model that comprised the 3D Resnet model, which effectively refined the important features. In addition, the channel and spatial attention techniques minimized the computational cost and time. However, the focal loss could mitigate the data imbalance problem. Even though the right steps were taken to improve the data, the model also required more data as the network got deeper to expand the model’s capabilities.

A multi-task learning model was utilized Liu et al. (2021), which identified the size of the nodule by using the anchor-free method. The model did not need the troublesome anchor-based strategy, the anchor-free method is more reliable and simpler to implement. However, the approach has a higher rate of missed detections because of interference from numerous potential false positives outside of the lung area.

Chen et al. (2021) initiated a dense neural network that could get superior accuracy compared to other current techniques while dealing with varying experimental targets and input lung CT image pixel sizes. Since LDNNET did not employ semantic segmentation and location, it was simpler to set up and operate in real-world scenarios. However, the model necessitated more parameters for learning that increased the training time.

Ji et al. (2023) designed an improved DL model with an attention method that improved the model’s detection performance and reduced the medical image interference features. In addition, the pyramid pooling module obtained the multi-scale contextual information and detected the lung nodules effectively. However, the model found trouble in detecting lung nodules when they were partly or obscured by other nodules, which could result in missed diagnoses or false positives.

Naseer et al. (2023) initiated an Alex Net-Support Vector Machine model for nodule detection and modified U-Net for segmentation, which demonstrated improved outcomes for the precise and efficient diagnosis of lung tumor. Nevertheless, the model required high computational time and reduced the interpretability and generalizability of lung nodule detection tasks.

A Retina Net model was implemented by Mahum and AlSalman (2023) that utilized a dependable feature fusion-based approach that could combine various network layers and concurrently boost the semantic shallow layer for efficient prediction. Additionally, at every network level, a context-dilated module is integrated with a down-sampled fusion block, which enhances the network’s capacity to extract useful features. However, the model could not distinguish microscopic cancers from background tissues if there was a lot of clutter or noise in the images.

The faster R-CNN model was utilized by Nguyen et al. (2021) for lung nodule detection, which minimized the false positives. The mean-shift clustering technique effectively learned the intricate features from the training dataset. Although the approach improved nodule detection performance, there are still several issues. First off, there was still a small volume of data available for the model’s training and validation. CNN increases network complexity to improve performance.

A multi-orientation-based attention mechanism was developed by Saihood et al. (2023) that provided a great degree of flexibility in focusing on relevant data that was non-locally gathered from different nodule regions. Although the research showed encouraging advancements, their practical application in healthcare settings required that their clinical efficacy be verified on actual patient data.

Gugulothu and Balaji (2024) introduced lung nodule detection and classification with CT images based on hybrid deep learning (LNDC-HDL) techniques. The use of computed tomography demonstrates the efficiency and importance of the HDE-NN specific structure for detecting lung nodes on CT scans, increases sensitivity, and reduces false positives. However, the major constraint of the research is computational complexity.

Sebastian and Dua (2023) implemented a DL model for the detection of lung nodules. To identify every lung lesion in every CT scan as completely as feasible without resorting to forced consensus this procedure is designed. However, the model suffers from computational time.

UrRehman et al. (2024) presented a CNN model with a dual attention mechanism for effective lung nodule detection. The CNN model extracts informative features from the images, while the attention module incorporates both channel attention and spatial attention mechanisms to selectively highlight significant features. However, a small volume of data was available for the model’s training and validation.

Harsono et al. (2022) introduced the Transfer learning-enabled one stage detector model, I3DR-Net for the detection of lung nodules. In particular, the I3DR-Net approach integrated the I3D backbone, with RetinaNet and modified FPN framework over the small number of high-quality trainable CT images and reduced the training time. Moreover, this approach demonstrated that weight transfer learning from natural image datasets improves the detection performance as well as minimizes the training time.

Wankhade, and Vigneshwari (2023) developed the Hybrid Neural Network approach which integrated the 3D CNN and RNN to enhance the detection performance. In addition, the hybrid approach precisely determined the lung nodule count in the pulmonary region. However, additional research was required to validate the results and address the potential challenges, including obtaining adequate high-quality training data and enhancing the comprehensibility of DL models.

Xing et al. (2024) developed an improved ML model integrating the Fuzzy K-Nearest Neighbor (FKNN) for lung disease detection. In addition, the EMRFO algorithm was applied to choose the optimal feature subset, and the FKNN model was utilized as the fitness evaluator. More specifically, the EMRFO algorithm presents the position information of the suboptimal solution in order to continuously approach the optimal solution with disturbance, thus improving the convergence of the algorithm.

### 2.2 Challenges

➢ The R-CNN architectures could face difficulties in precisely detecting borders, particularly for tumors with fuzzy or intricate features. Post-processing techniques are often necessary to improve border correctness (Nguyen, et al., 2021).
➢ Lung Retina-net is specifically engineered to identify lung tumors in CT scans. It might not be appropriate for different anatomical structures or other imaging modalities when it comes to detecting cancers (Mahum and AlSalman, 2023).
➢ Yolov5 struggles to discern cancers from intricate backgrounds, which might result in false positives or overlooked identifications in congested environments (Ji et al., 2023).
➢ Although focus loss was utilized to rectify data imbalance, it was recognized that its consequences might not be entirely eradicated. To mitigate this problem, more efforts were required (Zhang, et al., 2022).
➢ The AlexNet-SVM might not be easily interpreted, which could make it difficult to comprehend the rationale behind particular diagnoses. Additional verification, an evaluation of generalizability, and practical execution issues were required for clinical adoption (Naseer, et al., 2023).

## 3 Proposed CHSTM model for lung nodule detection

**FIGURE 1:**
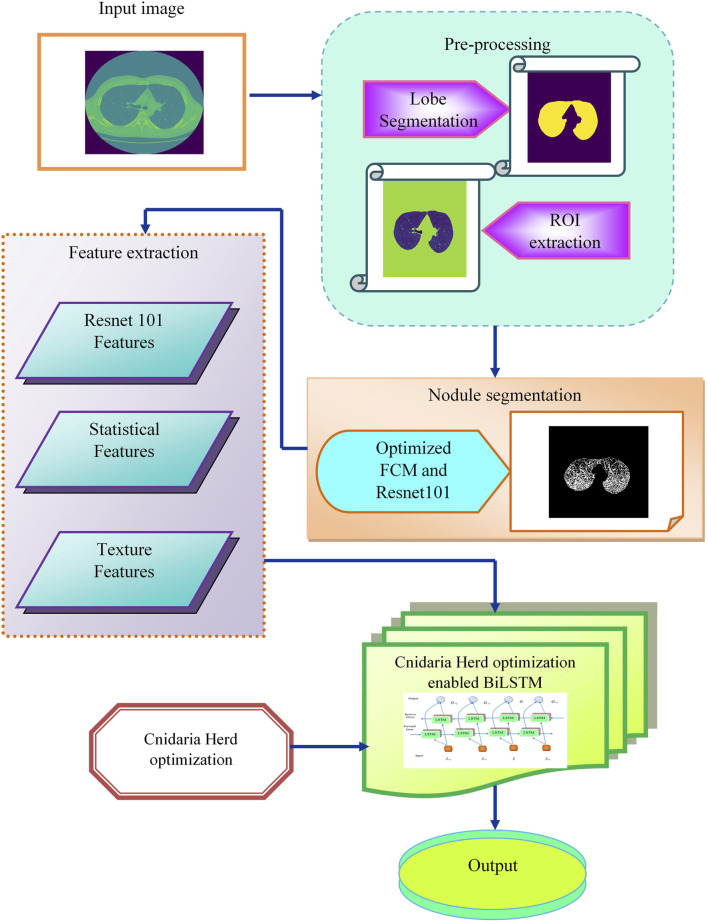
Flow diagram of the FC2R + CHSTM model for lung nodule detection.

Lung cancer diagnosis relies on detecting pulmonary nodules, but identifying small nodules in medical images can be challenging due to their varying shapes, sizes, and locations. Existing methods have limitations in scalability, robustness, data availability, and false detection rates. To address these issues, a novel deep-learning model is for accurate lung nodule detection. Initially the input CT images from the Luna 16 database undergo an image enhancement task in the pre-processing stage. The enhanced CT images are used to segment lung lobes, defining regions of interest (ROI). The pre-processing techniques used in lobe segmentation are erosion and fill holes. The segmented lobes are fed into the FC2R model for nodule segmentation. The proposed optimization is used to compute the coefficient features and the Resnet −101 model generates the flow maps that lead to improving the performance of the model. From the segmentation output, the Resnet −101 features, statistical features, and texture information are extracted which improve the nodule detection accuracy. These features are concatenated and input into the CHSTM classifier, which accurately detects lung nodules. The CHO algorithm which is inspired by jellyfish and krill fish characteristics, employed to fine-tune the hyperparameters of the model for optimal results. The combination of the induced movement strategy of krill with the time control mechanism of the cnidaria improves the CHSTM model’s performance to attain better detection accuracy. The proposed model aims to improve accuracy in detecting lung nodules from CT images and detect the lung nodules as malignant or benign. Figure 1 visualizes the flow diagram of this proposed approach.

### 3.1 Input for lung nodule detection

The Luna 16 database serves as the input source of this research, which consists of lung CT scans with the size of $\left(\;1*512*512\right)$ . The input for lung nodule detection is mathematically expressed in Equation 1 as follows,

$$L=\left\{l_{1},l_{2},.....l_{i},....l_{N}\right\}$$ (1)

where L specifies the dataset and the number of CT scans in the database is represented as $\left\{l_{1},l_{2},.....l_{i},....l_{N}\right\}$ .

### 3.2 Pre-processing

Pre-processing is a significant stage in image processing tasks, in this research it is employed to enhance the input image quality. In addition, lobe segmentation and ROI extraction are performed in this stage to improve the visibility of the image and improve the detection accuracy.

#### 3.2.1 Lobe segmentation

The lungs are made up of five lobes, split by the lobar fissures, with three lobes in the right lung and two in the left. The lobes receive blood and oxygen from different blood arteries, they are typically functionally independent. Moreover, illnesses frequently cannot spread between lobes because of the fissure border (Ferreira et al., 2018). To improve the detection accuracy of the CHSTM model, the lung lobes can be precisely segmented. The pre-processing techniques used in lobe segmentation are erosion and fill holes. The lobe segmentation in this research can be carried out in the following ways:

Step 1: Transform the CT image into a binary image
Step 2: Eliminate the blobs that are connected to the image’s border.
Step 3: Label the image.
Step 4: Keep the labels with the two largest regions
Step 5: Using a disk with a radius of two, do erosion. During this procedure, the lung nodules that are linked to the blood vessels are separated.
Step 6: Closure operation utilizing a 10-radius disk. The goal of this procedure is to maintain nodules affixed to the lung wall.

The technique held up well to varying inspiration levels, low-quality images, and varying disease severity, which makes it possible to do reliable and effective regional analysis on big datasets (Gerard and Reinhardt, 2019). The output of lobe segmentation $S_{l^{*}}$ is transferred into the ROI extraction process.

#### 3.2.2 Region of interest extraction

ROI extraction is the procedure of finding the parenchyma mask that may contain suspect lung nodules which reduces the system’s computation and detection times (Ozekes and Osman, 2010). The key objective of this process is to precisely evaluate lung images and extract the pertinent nodule regions while eliminating the background regions. The pre-processing technique used in ROI extraction is fill holes. In this research, the ROI extraction is mathematically defined in Equation 2 as follows,

$$R=l_{i}\left(∼\;S_{l^{*}}\right)$$ (2)

The output obtained from ROI extraction $\left(R\right)$ is provided in the FC2R model for nodule segmentation.

### 3.3 Cnidaria herd optimization algorithm for lung nodule detection

#### 3.3.1 Motivation

The CHO algorithm mimics the food-searching behaviour of the cnidaria and the herding features of the krill. The standard algorithms maintain a positive balance between local and global searches and have a solid population usage rate. However, the algorithm’s speed of convergence and capacity for exploration and exploitation is less. In addition, the algorithms require an optimal strategy to initiate the distribution process. To address these issues, the research presented a novel CHO algorithm that enhances the model’s performance and increases detection accuracy by fusing the induced movement strategy of krill with the temporal control mechanism of cnidaria.

#### 3.3.2 Inspiration

Jellyfish also known as cnidaria can be found in water with a range of temperatures and depths, which resemble the bell-like structure and have unique characteristics that allow them to regulate their motion. To move their bodies forward, their undersides close like an umbrella, forcing water out. Even with this ability, they primarily float on the water, meaning that tides and currents control their movement. Specifically, cnidaria are weak swimmers, their orientations concerning currents are particularly important for maintaining blooms and preventing stranding. A swarm’s development is influenced by a variety of elements, such as temperature, predator presence, oxygen availability, available nutrition, and accessible nutrients. Ocean currents are the most significant of them since they can gather cnidaria into swarms. Because they tended to swarm, move about within swarms, and generate blooms as they follow ocean currents. As a result, cnidaria evaluates the quantity of food offered at several locations and chooses the finest one (Chou and Molla, 2022). The krill use a food search strategy, which involves locating places with large concentrations of food, and density-dependent attraction, to move toward the global minima. In this method, each krill looks for the highest density and food, and it travels in the direction of the best solution. In other words, the objective function decreases with increasing distance from the high density and food. For a single goal, it is generally necessary to define some coefficients when employing multi-objective herding behavior (Gandomi and Alavi, 2012). The hybrid algorithm solves the global optimization problems and tunes the hyperparameters of the CHSTM technique for precise detection of lung nodules.

##### 3.3.2.1 Initialization

The solution’s population is normally initialized as random; the solution’s position is mathematically formulated in Equation 3 as follows,

$$B_{t}=B_{t-1}-r_{1}\left(U_{up}-U_{low}\right)V_{t-1}$$ (3)

where $B_{t}$ indicates the solution’s current position, $B_{t-1}$ denotes the previous position of the solution, $V_{t-1}$ signifies the velocity of the solution at the previous iteration, and $r_{1}$ indicates the positional factor, the lower and upper bounds are denoted as $U_{low}$ and $U_{up}$ .

##### 3.3.2.2 Fitness evaluation

The solution’s fitness can be assessed using Equation 4, the higher fitness value indicates the best solution and the lower value denotes the worst solution.

$$F\left(B_{t}\right)=\max\left(Accuracy\left(B_{t}\right)\right)$$ (4)

Phase 1: Exploration phase $rand\left(0,1\right)>\left(1-\zeta \left(t\right)\right)$

When the random simulated movement of the solution is superior to the time control function the exploration phase is activated. The exploration phase describes that the solution should be in motion towards ocean currents. At this phase, the solution includes its foraging behaviors in a wide range around the search space. In terms of two primary effective parameters, the foraging movement towards the ocean current is formulated. The location of the food is the first element, and previous knowledge of the place is the second. Thus, the foraging behavior as its passive motion towards the search space has been mathematically formulated in Equation 5 as,

$$B_{t+1}=\frac{V_{F}\alpha_{i}+\varpi_{F}\left(B_{t}\right)+B_{t}+r_{3}\left(B_{G}-a\mu \right)}{2}$$ (5)

where, $rand\left(0,1\right)$ denotes the random simulated movement of the solution, and $\zeta \left(t\right)$ indicates the time control function, which is explained in Equation 6 as,

$$\zeta \left(t\right)=\left(1-\frac{t}{t_{\max}}\left(2r_{2}\left(0,1\right)-1\right)\right)$$ (6)

here, $r_{2}$ signifies the hectic factor, the recursive factor is indicated as $r_{3}\in \left(0.1,1\right)$ , μ represents the mean position of all solutions in the swarm, a signifies the trial factor, $V_{F}$ is the foraging speed factor, $B_{G}$ signifies the global best solution, the adaptive weightage factor is denoted as $\varpi_{F}$ , the differential fitness factor $\alpha_{i}$ is described in Equation 7 as follows,

$$\alpha_{i}=F\left(B_{t}\right)-F\left(B_{G}\right)$$ (7)

where $F\left(B_{t}\right)$ indicates the fitness value of the solution, $F\left(B_{G}\right)$ is the fitness function of the global best solution.

Phase 2: Exploitation phase $rand\left(0,1\right)<\left(1-\zeta \left(t\right)\right)$

In the exploitation phase, the solution exhibits movement inside the swarm behavior (active motion), and this movement of the solutions is considered as the exploitation phase. This phase is achieved by using the induced movement behavior of the solutions in which the individual solutions try to conserve a high density and move due to their mutual effects. Thus the solution obtains safety search inside the swarm and increased efficiency while maintaining inside the swarm is expressed in Equation 8 as follows,

$$B_{t+1}=\frac{\left(B_{t}+\gamma r_{4}\left(U_{up}-U_{low}\right)\right)+\left(B_{G}\varphi_{B}+\varpi_{n}B_{t-1}\right)}{2}$$ (8)

where γ denotes the sensitive factor, $r_{4}$ represents the cooperative movement behavior of solutions, and the effective factor $\varphi_{B}$ is the combination of the local effect $\left(\varphi^{local}\right)$ and the target direction effect $\left(\varphi^{t\arg et}\right)$ which is mathematically explained in Equation 9 as follows,

$$\varphi_{B}=\varphi^{local}+\varphi^{t\arg et}$$ (9)

The effect of the neighboring solutions in a movement can be evaluated in Equation 10 as follows,

$$\varphi^{local}=\sum_{t=1}^{n}{\hat{F}}_{t}\hat{B}$$ (10)

where $\hat{B}$ indicates the adaptive positional vector, the adaptive fitness vector ${\hat{F}}_{t}$ is calculated using the Equations 11, 12.

$${\hat{F}}_{t}=\frac{F\left(B_{t}\right)-F\left(B_{t-1}\right)}{F\left(B_{worst}\right)-F\left(B_{G}\right)}$$ (11)

$$\hat{B}=\frac{B_{t-1}-B_{t}}{\left\|B_{t-1}+B_{t}\right\|+\varepsilon }$$ (12)

where $B_{worst}$ represents the worst solution, and ε denotes the small positive number.

Phase 3: Equilibrium phase $rand\left(0,1\right)=\left(1-\zeta \left(t\right)\right)$

**FIGURE 2:**
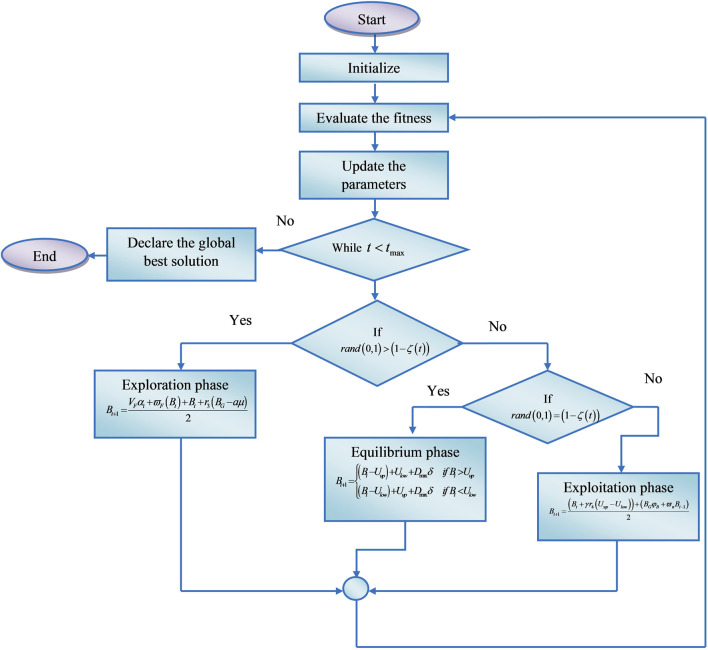
Flow diagram of the CHO algorithm.

In the equilibrium phase, there is a balance between exploration and exploitation, thus the solution effectively achieves equilibrium between these two phases also the physical diffusion process fastens the change in the equilibrium described in adaptive fitness vector is calculated in Equation 13 as follows,.

$$B_{t+1}=\left\{\begin{array}{l} \left(B_{t}-U_{up}\right)+U_{low}+D_{\max}\delta \;\mathrm{if}\;B_{t}>U_{up}\\ \left(B_{t}-U_{low}\right)+U_{up}+D_{\max}\delta \;\mathrm{if}\;B_{t}<U_{low}\{ \end{array}\right.$$ (13)

where the maximum diffusion speed is represented as $D_{\max}$ , and δ indicates the directional factor. Finally, when the algorithm reaches its termination condition the iteration will be stopped. For increased detection accuracy, the CHSTM classifier’s hyperparameters are efficiently adjusted using the suggested CHO method. Figure 2 displays the CHO algorithm’s flow diagram.

### 3.4 Nodule segmentation using fuzzy C-means clustering enabled Resnet −101

Accurate segmentation of lung nodules from CT images is an essential task for image-driven lung nodule detection. Robust nodule segmentation is difficult, nonetheless, because lung nodules vary widely, and similar visual traits are common to lesions and their surroundings (Wang et al., 2017). Therefore, this research proposed an FC2R segmentation model which is the combination of a fuzzy C-means clustering algorithm and Resnet −101 deep learning model. The process of grouping data into homogenous units by taking object relationships into account is known as clustering. Clustering techniques accomplish region segmentation by dividing the image into groups of pixels that have a high degree of similarity within the feature space. In fuzzy clustering, individual data components have a set of membership levels associated with them and can belong to many clusters. These display the degree to which a given data element is linked to a certain cluster (Nithila and Kumar, 2016). The process of assigning the membership levels to allocate data components to single or more clusters is known as fuzzy clustering. Let $R=\left\{h_{1},h_{2},......h_{N}\right\}$ be the ROI extracted image, the cluster centers $C=\left\{d_{1},d_{2},......d_{C}\right\}$ along with the partition matrix are denoted as $=w_{ik}\in \left[0,1\right]$ . The degree to which an instance $h_{k}$ belongs to the cluster $d_{i}$ is determined by each component $w_{ik}$ . The objective function for FCM is derived in Equation 14 as follows (Farahani et al., 2018),

$$D\left(W,C\right)=\sum_{i=1}^{c}\sum_{k=1}^{n}w_{ik}^{x}{\left\|h_{k}-d_{i}\right\|}^{2}$$ (14)

where, each component and cluster are described in Equations 15, 16 as follows,

$$w_{ik}=\frac{1}{\sum_{i=1}^{c}{\left(\frac{\left\|h_{k}-d_{i}\right\|}{\left\|h_{k}-d_{i}\right\|}\right)}^{\frac{2}{\left(x-1\right)}}}$$ (15)

$$d_{i}=\frac{\sum_{k=1}^{n}w_{w_{ik}}^{x}h_{k}}{\sum_{k=1}^{n}w_{w_{ik}}^{x}}$$ (16)

Where x signifies the fuzzier which indicates the cluster fuzziness level, The FCM cluster aims to lower the total weighted mean square error. Every feature vector is approved by the FCM to correspond with several groups with diverse membership values (Ganesan and Merline, 2017). The segmented image with the dimension of $\left(1*230*230*3\right)$ is provided as input to the feature extraction process.

### 3.5 Feature extraction

Feature extraction is the process of distilling relevant information from the segmented image. It aims to extract a concise representation, often expressed as numerical descriptors or attributes. In the context of lung nodule detection, it helps to identify relevant patterns and characteristics. The following features are extracted from segmented lung nodule images.

#### 3.5.1 Resnet −101 feature extraction

Resnet −101 is a variation of the deep CNN model which comprises 101 layers along with the skip connections. The basic idea behind residual functions is that each layer of the network learns from them by referring to its input layer (Ali et al., 2021). The architecture is easily optimized in this way and achieves notable accuracy. The network’s depth is based on the internal convolutional layers. The implementation of Resnet −101 in this research improves both the model’s detection accuracy and feature extraction capacity. The features obtained using Resnet −101 with the size of $\left(1*1024\right)$ are denoted as $\left(f_{R}\right)$ .

#### 3.5.2 Statistical features

The numerical measurements used to depict the distributional characteristics of geometric properties within specific regions are evaluated using statistical features. These characteristics improve the model’s performance by providing significant insights into the statistical characteristics of lung nodule images (Mutlag et al., 2020). In this research features including kurtosis $\left(K_{R}\right)$ ,standard deviation $\left(\sigma_{R}\right)$ mean $\left(M_{R}\right)$ , skewness $\left(S_{R}\right)$ , and variance $\left(\nu_{R}\right)$ are extracted from the segmented image. The standard deviation is used to calculate the mean distance between the pixel values, while the mean measures the average of the pixel intensities. Kurtosis can be used to assess the distribution’s stability, and skewness is a measure of the distribution’s asymmetry. The extracted features with the size of $\left(1*5\right)$ are concatenated as $st_{R}=\left\|M_{R}\right\|K_{R}\left\|S_{R}\right\|\sigma_{R}\left\|\nu_{R}\right\|$ .

#### 3.5.3 Texture features

Texture is a significant component in medical images, which is described as a surface-level representation of the human visual systems. The texture features are analysed using matrix representations. In this research, the texture features are analysed using the local Ternary pattern (LTP) and local optimal oriented pattern (LOOP). The LTP features address the issues related to illumination transformation, which is robust to noise and encodes the grey values of surrounding pixels. The LTP indicator ltp is described in Equation 17 as follows, (Wu et al., 2015)

$$ltp=\sum_{n=0}^{P-1}b\left(J_{n}-J_{c}\right).3^{n}b\left(A\right)=\left\{\begin{array}{l} 1A\geq \tau \\ 0-\tau <A<\tau \\ -1A<-\tau \end{array}\right.$$ (17)

where τ indicates the user-defined threshold, the central pixel’s gray value is signified as $J_{c}$ and neighbouring pixels’ gray value is indicated as $J_{n}$ . LOOP is the non-linear fusion of binary and directional patterns, which offers several advantages in image processing tasks. Let $\left(m_{c},n_{c}\right)$ be the pixel values of a segmented image A , the pixel intensity is indicated as $I_{N},N=0,...7$ , based on the rank of magnitude value of the exponential $\left(u_{N}\right)$ is assigned to each pixel (Chakraborti et al., 2018). Further the LOOP descriptor is derived based on the Equation 18 as follows,

$$loop\left(m_{c},n_{c}\right)=\sum_{N=1}^{7}b\left(I_{N}-I_{c}\right)2^{u_{N}}$$ (18)

where the threshold function is explained in Equation 19 as follows,

$$b\left(A\right)=\left\{\begin{array}{l} 1\;if\;A\geq 0\\ 0\;otherwise \end{array}\right.$$ (19)

The LOOP descriptor effectively determines the rotation invariance, which also reverses the empirical assignment of the value. The extracted feature vectors using Resnet-101 features $\left(1*1024\right)$ , statistical features $\left(1*5\right)$ and texture features $\left(1*32*32\right)$ are concatenated as $E=\left\|f_{R}\right\|st_{R}\left\|T_{R}\right\|$ , with the size of $\left(1*1024\right)$ which is presented into the CHSTM model.

### 3.6 Lung nodule detection using cnidaria herd optimization -enabled Bi-directional long short-term memory model

**FIGURE 3:**
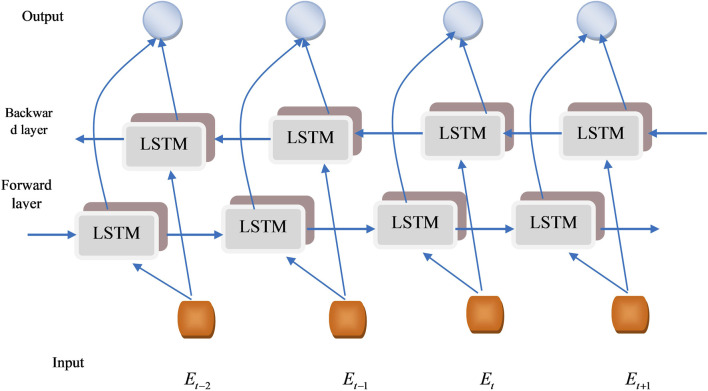
BiLSTM architecture.

The research presented a novel CHSTM model for lung nodule detection; an input gate, a memory gate, and an output gate are the three gate control units that the proposed CHSTM model in this research uses to enhance memory capability and efficiently detect lung nodules. The architecture of the single BiLSTM block is visualized in Figure 3. Neurons in the CHSTM are capable of selectively forgetting and recalling new information about the cell state through the forgetting and memory gates. They can also retain this important information and send it on to subsequent neurons. The input through the neuron’s internal weight computation controls the forgetting, remembering, and output of the information. The weights of the gate control units are obtained from the previous moment. The detection accuracy of the model is improved by CHSTM’s ability to fully account for both precedent and upcoming data more easily than LSTM (Hameed and Garcia-Zapirain, 2020). The equations of the CHSTM model is shown in Equations 20–24.

$$Q_{t}=\sigma \left(\omega_{Q}.\left[s_{t-1},E_{t}\right]+d_{Q}\right)$$ (20)

$$I_{t}=\sigma \left(\omega_{I}.\left[s_{t-1},E_{t}\right]+d_{I}\right)$$ (21)

$$P_{t}=\sigma \left(\omega_{P}.\left[s_{t-1},E_{t}\right]+d_{P}\right)$$ (22)

$${\overset{˘}{X}}_{t}=\tan h\left(\omega_{X}.\left[s_{t-1},E_{t}\right]+d_{X}\right)$$ (23)

$$X_{t}=Q_{t}⊙X_{t-1}+I_{t}⊙{\overset{˘}{X}}_{t}$$ (24)

$$s_{t}=P_{t}⊙\tan h\left(X_{t}\right)$$ (25)

where the input is signified as $I_{t}$ , $Q_{t}$ and $P_{t}$ indicates the input, output, and forget gate respectively. σ and tanh denotes the activation functions, $X_{t}$ represents the cell state, and the weights and biases of the gates are denoted as $\omega_{Q},\omega_{I},\omega_{P},\omega_{X}$ and $d_{Q},d_{I},d_{P},d_{X}$ , which are optimized using the proposed CHA optimization. The element-wise multiplication is denoted as ⊙ , and $s_{t}$ signifies the hidden layer state. The output from the feature extraction is concatenated and fed as the input to the CHSTM model which helps to accurately detect the lung nodules. The CHO algorithm is applied to tune the model weights for optimal results.

By reducing the vanishing gradient issue, multiplicative gates enable memory cells to store and retrieve data for extensive periods. The activation of the cell state is preserved using the input gate. Because of this feature, the network may store crucial information from previous sequences that enhances the detection performance (Elzayady et al., 2021). BiLSTM can learn more precise information by using the information’s forward and backward sequences (Ganesan and Merline, 2017). Three forward $\left({\vec{s}}_{t}\right)$ and backward LSTM $\left({\overset{⃖}{s}}_{t}\right)$ layers create the CHSTM model, and the fully connected layer receives the output from these layers. The forward LSTM processes information from left to right and the backward LSTM processes information from right to left (Bhanumathi and Chandrashekara, 2021). The output of the CHSTM model is computed as $s_{t}=\left[{\vec{s}}_{t},{\overset{⃖}{s}}_{t}\right]$ , where the output of forward gate and backward gate is expressed in Equations 26, 27 respectively as follows,

$$\vec{s_{t}}=lstm\left(E_{t},s_{t-1}^{\to }\right)$$ (26)

$${\overset{⃖}{s}}_{t}=lstm\left(E_{t},s_{t+1}^{\to }\right)$$ (27)

**FIGURE 4:**
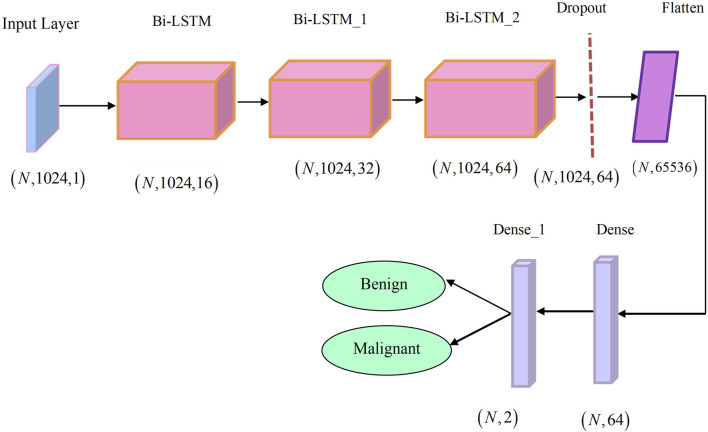
Architecture of the CHSTM model.

The CHSTM model’s final layer, the linear regression layer, completes regression tasks that help to detect lung nodules. The architecture of the CHSTM model is illustrated in Figure 4.

## 4 Results and discussion

The following explains the findings and analysis of the suggested model, along with a performance assessment and comparison analysis.

### 4.1 Experimental setup

The present investigation utilized data acquired from the LUNA 16 dataset consisting of 888 CT scan samples. The performance of the model is evaluated with TP and K-fold by varying epochs. The dataset was partitioned into 80% of training data and 20% of testing data. The model is trained on the training set and the performance is evaluated on the testing set. The research lung nodule detection using FC2R + CHSTM is executed in PYTHON software with Windows 11 operating system, 16 GB RAM, 1 TB ROM, AMD RYZEN 5000H Series, and including GPU. The hyperparameters of the model includes the learning rate of 0.001, epoch of 100, batch size of 64, and the ADAM as the default optimizer.

### 4.2 Dataset description

#### 4.2.1 LUNA 16 dataset (Kaggle datasets, 2024)

LUNA 16 is a publicly available LIDC/IDRI dataset and is a collaborative effort between the Lung Imaging Database Consortium (LIDC) and the Image Database Resource Institute (IDRI). Comprising 888 CT scans, this publicly accessible medical imaging dataset focuses on lung cancer research. It includes both malignant and benign nodules, enabling scientists to assess the performance of algorithms in distinguishing between these two types of nodules. Further, the radiologists annotated the lesions as non-nodule, nodules <3 mm, and nodules ≥3 mm.

#### 4.2.2 LIDC/IDRI dataset

This research utilized the LIDC/IDRI data set [10], comprising 888 thoracic CT scans with a section thickness of 2.5 mm or lower. Further, the dataset shows the influence of the presence of contrast, section thickness, and reconstruction kernel for assessing the computer-aided detection (CAD) performance. Four radiologists independently scrutinized the false positive CAD marks of the best CAD system.

### 4.3 Performance metrics

The performance evaluation of the proposed FC2R + CHSTM model involves assessing key metrics, such as sensitivity accuracy, and specificity. Accuracy represents the ratio of correctly identified lung nodules to the total number of detected nodules using the FC2R + CHSTM model. Sensitivity is defined as the fraction of lung nodule instances that are positive. Specificity gauges how well the model can distinguish non-tumor areas from the CT scan.

### 4.4 Experimental results

**FIGURE 5:**
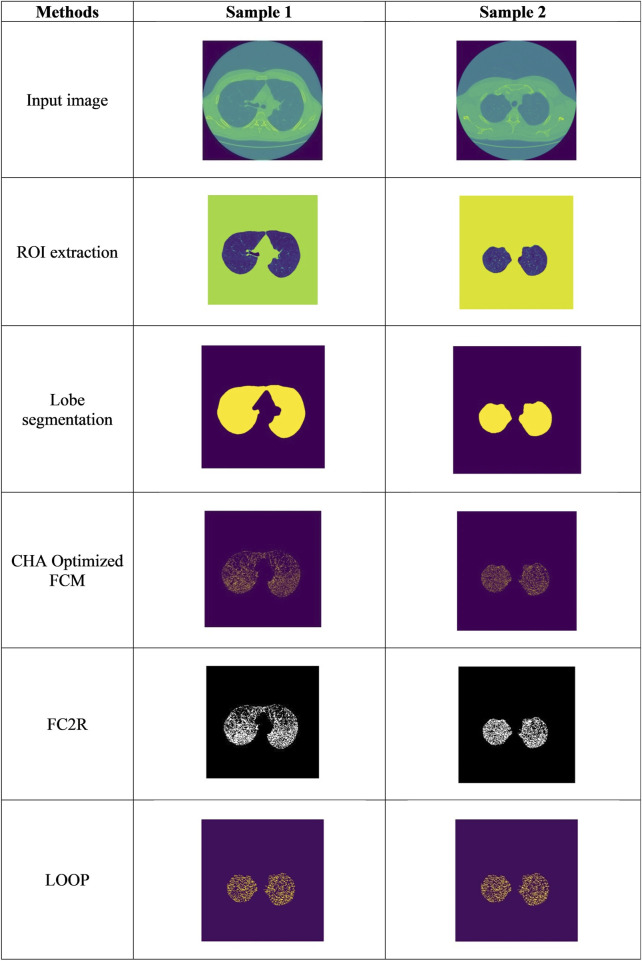
The experimental results obtained from the FC2R+CHSTM model.

The experimental outcomes obtained from the FC2R + CHSTM model are depicted in Figure 5, which also illustrates the input CT image, the pre-processing technique such as ROI extraction output, and lobe segmentation outcomes. The final outcome of the Sample 1 denotes the lung nodules are benign and sample 2 denotes the lung nodules are malignant.

### 4.5 Performance analysis

#### 4.5.1 Analysis of performance with TP

**FIGURE 6:**
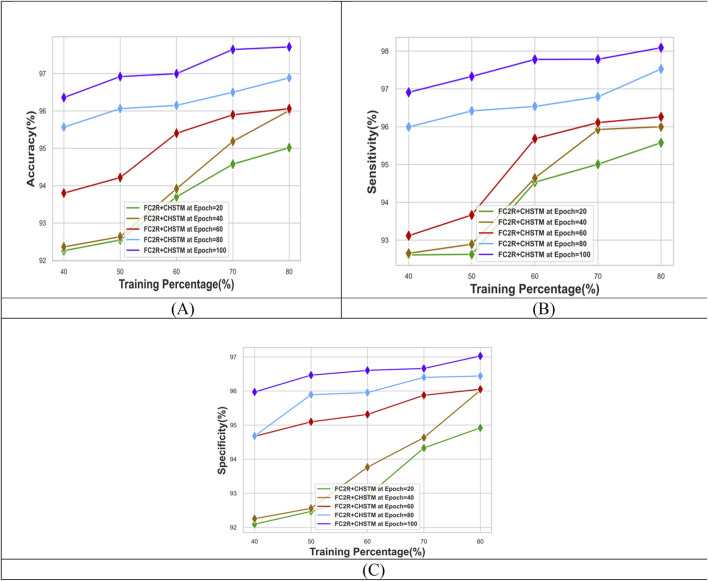
Performance evaluation with TP showing **(A)** Accuracy, **(B)** Sensitivity and **(C)** Specificity.

The performance evaluation for the FC2R + CHSTM model in terms of Training percentage (TP) analysis is shown in Figure 6. At TP 80 and epoch 40 the FC2R + CHSTM attains 96.00% accuracy as well as for epoch 100 the accuracy of the model is 97.71%. Similarly, for epoch 100 and TP 80, the FC2R + CHSTM model attains 98.08% sensitivity. The specificity value attained by the proposed FC2R + CHSTM model is 97.02% which demonstrates that the FC2R + CHSTM model detects the lung nodules with improved performance. The results depict that the model’s accuracy is improved by increasing the size of the epoch. The hybridization of the BiLSTM classifier with the CHA optimization increases the convergence of the model to get the finest results. Additionally, the FC2R segments the nodule regions with higher accuracy which minimizes the computational complexity.

#### 4.5.2 Analysis of performance with K-fold

**FIGURE 7:**
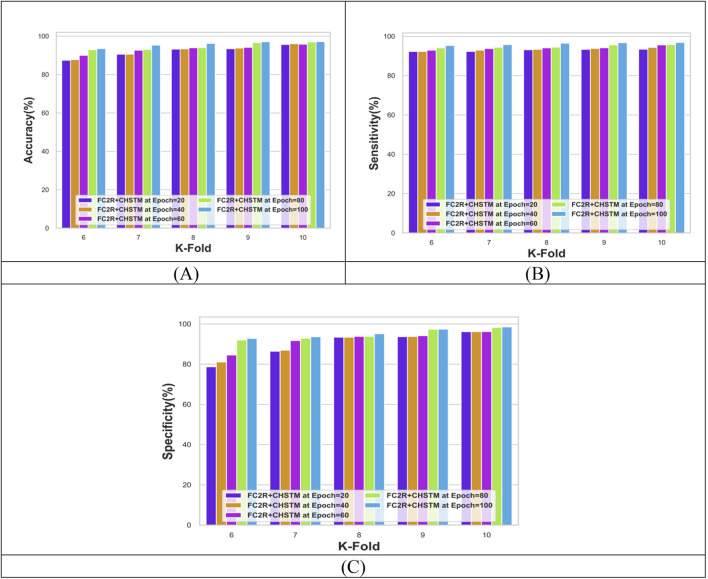
Performance evaluation with K-fold showing **(A)** Accuracy, **(B)** Sensitivity and **(C)** Specificity.

Figure 7 shows the performance analysis of the FC2R + CHSTM model with k-fold analysis for various epochs. For epoch 100, the model’s accuracy is 97.15%, while at k-fold 10 and epoch 40, the FC2R + CHSTM achieves 96.07% accuracy. As the epoch size increases, the model’s accuracy increases as well, according to the data. Similarly, the FC2R + CHSTM model achieves 96.87% sensitivity for k-fold 10 and epoch 100. The proposed FC2R + CHSTM model attains a specificity value of 98.53%, indicating that it performs better in detecting lung nodules. To achieve optimal results, the hybridization of the BiLSTM classifier with the CHA optimization improves the model’s convergence. In addition, the FC2R segments the nodule regions with higher accuracy which minimizes the computational complexity.

### 4.6 Comparative techniques

The performance of the FC2R + CHSTM model is compared with other conventional models such as Salmon fish optimization (SFO Based Deep NN) (Mozaffari and Fathi, 2012), Support Vector Machine (SVM) (Khan et al., 2019), Convolutional Neural Network (CNN) (Huang et al., 2017), Deep Neural Network (Deep NN) (Shukla et al., 2021), Artificial Neural Network (ANN) (Dandıl et al., 2014), K-Nearest Neighbour (KNN) (Lennartz et al., 2021), BiLSTM (Ganesan and Merline, 2017), Krill herd optimization (KHO Based Deep NN) (Yu et al., 2022), Jellyfish optimization based BiLSTM (JFOSTM) (Chou and Molla, 2022), Krill herd optimization enabled BiLSTM (KHOSTM) (Gandomi and Alavi, 2012), SKO based Deep NN, LNDC-HDL (Gugulothu and Balaji, 2024), and CNN + IMFO + LBP (Sebastian and Dua, 2023), I3DR-Net (Harsono et al., 2022), Modified AlexNet-SVM (Naseer et al., 2023) to explicate the efficiency of the model in lung nodule detection.

#### 4.6.1 Comparative evaluation with TP utilizing LUNA 16 dataset

**FIGURE 8:**
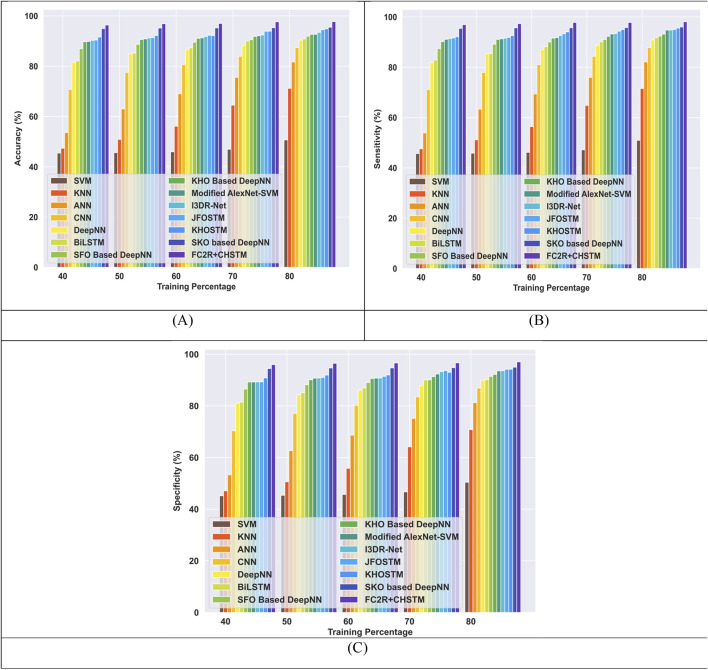
Comparative evaluation with TP using LUNA 16 dataset. **(A)** Accuracy, **(B)** Sensitivity and **(C)** Specificity.

Figure 8 presents a comparison of the FC2R + CHSTM with the traditional approaches using TP for the Luna 16 dataset using TP analysis. The FC2R + CHSTM model achieves 97.71%, outperforming the current CNN by 10.60%, BiLSTM by 7.03%, Modified AlexNet-SVM by 5.16%, and I3DR-Net by 4.38%. For sensitivity measure, the model attains 98.08% which shows improvement over the Modified AlexNet-SVM by 3.41%, I3DR-Net by 3.34%, and JFOSTM by 3.16%. For TP 80 the FC2R + CHSTM obtained 97.02% specificity that demonstrates superior performance improvement than the conventional Modified AlexNet-SVM, I3DR-Net, and JFOSTM by 3.57%, 3.50%, and 2.93% respectively. Thus, the FC2R + CHSTM model obtains enhanced results than the existing methods, and the integration of the CHO algorithm combines the induced movement strategy of krill with the time control mechanism of the cnidaria that increases the performance of theFC2R + CHSTM model to attain better detection accuracy.

#### 4.6.2 Comparative evaluation with K-fold utilizing LUNA 16 dataset

**FIGURE 9:**
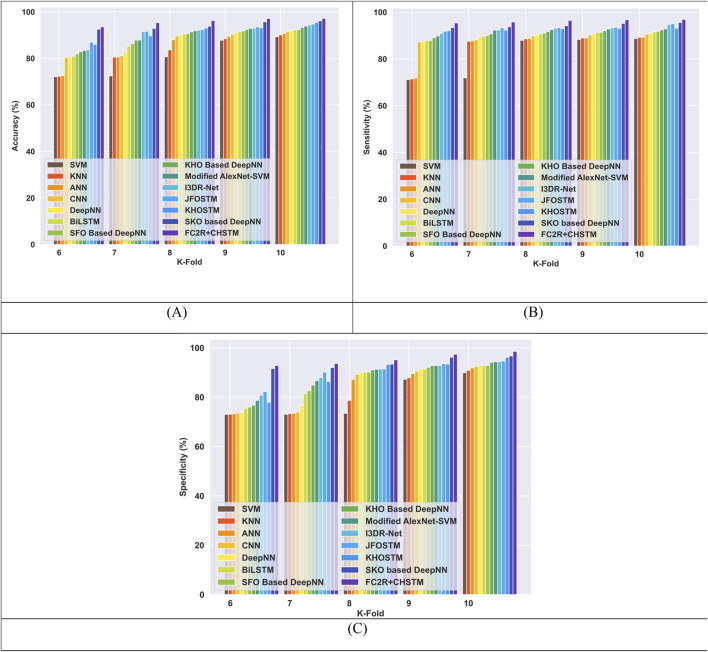
Comparative evaluation with k-fold using LUNA 16 dataset. **(A)** Accuracy, **(B)** Sensitivity and **(C)** Specificity.

Figure 9 compares the FC2R + CHSTM model performance to the existing methods using K-fold 10. The FC2R + CHSTM model obtains 97.15%, outperforming the Modified AlexNet-SVM by 3.41%, I3DR-Net by 2.88%, and JFOSTM by 2.47%. For sensitivity measure, the model attains 96.87% which exceeded the existing technique Modified AlexNet-SVM by 4.18%, I3DR-Net by 2.22%, and JFOSTM by 1.90%. For K-fold 10 the FC2R + CHSTM obtained 98.53% specificity that demonstrates superior performance improvement than the conventional Modified AlexNet-SVM, I3DR-Net, and JFOSTM by 4.24%, 4.18%, and 3.91% respectively. Thus, the FC2R + CHSTM model obtains enhanced results than the implemented methods, and the integration of the CHO algorithm combines the induced movement strategy of krill with the time control mechanism of the cnidaria that advances the performance of the FC2R + CHSTM model.

#### 4.6.3 Comparative evaluation with TP utilizing LIDC/IDRI dataset

**FIGURE 10:**
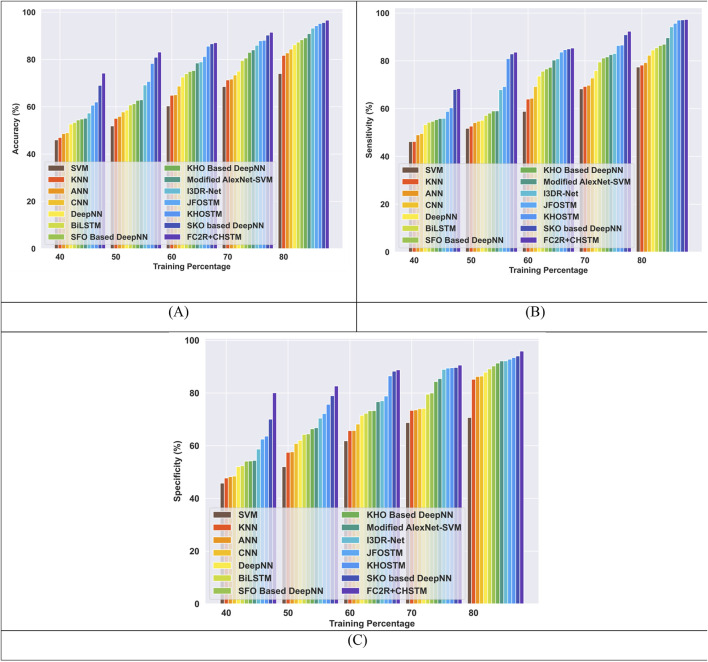
Comparative evaluation with TP using LIDC or IDRI dataset. **(A)** Accuracy, **(B)** Sensitivity and **(C)** Specificity.

The comparative evaluation of the FC2R + CHSTM and the conventional methods utilizing TP for the LIDC/IDRI dataset is shown in Figure 10. The FC2R + CHSTM model outperforms the Modified AlexNet-SVM by 5.87%, I3DR-Net by 3.50% and JFOSTM by 2.43%, with a high accuracy of 96.60%. With a sensitivity measure of 97.30%, the proposed model outperforms the Modified AlexNet-SVM by 7.83%, I3DR-Net by 3.15%, and JFOSTM by 1.65%. In comparison to the traditional Modified AlexNet-SVM, I3DR-Net, and JFOSTM, the proposed approach attained a significant improvement of 3.88%, 3.85%, and 3.22% better, respectively, achieving a specificity of 95.90% for TP 80. More specifically, the FC2R + CHSTM model outperforms the other existing techniques via the application of the CHO algorithm combines the cnidaria’s time control mechanism with the krill’s induced movement strategy, improving the FC2R + CHSTM model’s performance and achieving higher detection accuracy.

#### 4.6.4 Comparative evaluation with K-fold utilizing LIDC/IDRI dataset

**FIGURE 11:**
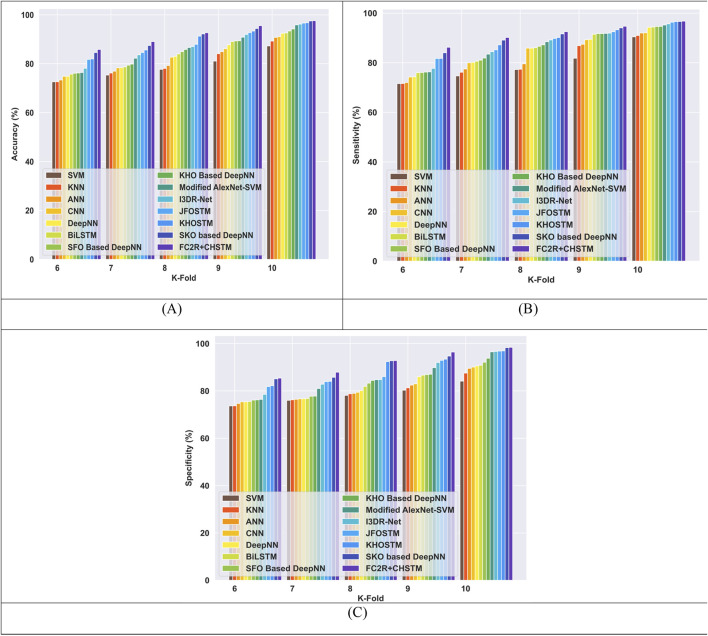
Comparative evaluation with k-fold using LIDC or IDRI dataset. **(A)** Accuracy, **(B)** Sensitivity and **(C)** Specificity.

The performance of the FC2R + CHSTM model is compared to the existing approaches in terms of K-fold 10 with the LIDC/IDRI dataset in Figure 11. The FC2R + CHSTM model achieves 97.59%, surpassing the Modified AlexNet-SVM by 1.77%, I3DR-Net by 1.45% and JFOSTM by 1.04%. With a sensitivity measure of 96.77%, the FC2R + CHSTM model outperforms the Modified AlexNet-SVM by 1.61%, I3DR-Net by 1.14%, and JFOSTM by 0.45%. Subsequently, the FC2R + CHSTM achieves 98.30% specificity for K-fold 10 and obtained a substantial improvement of 1.92% over AlexNet-SVM,1.76% over I3DR-Net, and 1.62% over JFOSTM. The CHO algorithm integrates the induced movement strategy of krill with the time control mechanism of cnidaria, improving the performance of the FC2R + CHSTM model. As a result, the model achieves better outcomes than the other existing approaches.

### 4.7 Comparative discussion

The implemented methods employed for lung nodule detection pose several limitations related to generalizability, interpretability challenges, and data requirements. In addition to that the conventional SVM model creates data imbalance issues which affect the reliability of the detection model. While the modified attention-based techniques mentioned in the literature minimized the computational cost, it has a higher rate of missed detections because of interference from numerous potential false positives outside of the lung area. The LNDC-HDL technique faces challenges in computational complexity. The CNN + IMFO + LBP model suffers from computational time. To overcome this issue this research proposed the FC2R + CHSTM model which detects the lung nodules with better accuracy and minimum run time. The CHSTM + FC2R model increases the system reliability minimizes the overfitting issues caused by data imbalance problems and increases the computational efficiency of the lung nodule detection tasks. The comparative discussion of the FC2R + CHSTM model concerning TP and K-fold analysis is shown in Table 1.

**TABLE 1:** Comparative analysis with the proposed model using the LUNA 16 dataset.

Methods	TP (80)			K-fold (10)		
	Accuracy (%)	Sensitivity (%)	Specificity (%)	Accuracy (%)	Sensitivity (%)	Specificity (%)
SVM	50.67	50.94	50.41	89.29	88.69	89.90
KNN	71.18	71.56	70.81	90.03	89.19	90.87
ANN	81.69	82.12	81.27	90.58	89.31	91.85
CNN	87.36	87.81	86.90	91.54	90.56	92.52
DeepNN	90.39	90.86	89.92	92.01	91.18	92.84
BiLSTM	90.85	91.76	90.12	92.21	91.55	92.85
SFO Based DeepNN	91.91	92.39	91.43	92.33	91.80	92.86
KHO Based DeepNN	92.67	93.15	92.18	93.28	92.43	94.13
Modified AlexNet-SVM	92.67	94.74	93.56	93.85	92.82	94.35
I3DR-Net	93.43	94.81	93.63	94.36	94.72	94.42
JFOSTM	94.49	94.99	94.18	94.76	95.03	94.68
KHOSTM	94.84	95.57	94.20	95.47	93.10	96.15
SKO based DeepNN	95.50	96.00	95.00	96.11	95.54	96.69
FC2R + CHSTM	97.71	98.09	97.03	97.16	96.87	98.53

### 4.8 Statistical analysis

Statistical Analysis is carried out on different datasets including the LUNA 16 dataset and LIDC/IDRI dataset to evaluate the robustness of the results. In addition, the statistical measures such as best, mean and variance are applied for evaluating the metrics accuracy, sensitivity, and specificity, and the results are described in this section. Tables 2, 3 reveal the results of the statistical analysis for the LUNA 16 dataset and LIDC/IDRI dataset respectively. Moreover, the statistical analysis portrayed in Tables 2, 3, ensures that the proposed research obtained robust results in terms of performance metrics facilitating the improved interpretation of results.

**TABLE 2:** Statistical analysis using the LUNA 16 dataset.

Methods	Accuracy			Sensitivity			Specificity		
	Best	Mean	Variance	Best	Mean	Variance	Best	Mean	Variance
SVM	74.05	60.17	106.36	77.41	60.49	126.09	70.70	59.84	91.88
KNN	81.70	63.99	147.32	78.20	62.07	130.98	85.20	65.92	165.76
ANN	82.75	64.80	142.00	79.23	63.29	117.02	86.27	66.32	170.36
CNN	84.38	66.67	150.73	82.33	65.74	143.80	86.42	67.60	161.69
DeepNN	86.22	69.01	144.26	84.54	68.49	149.74	87.90	69.54	145.06
BiLSTM	87.33	70.99	152.87	85.47	70.43	154.35	89.18	71.55	158.65
SFO Based DeepNN	88.35	71.93	154.76	86.44	71.41	160.90	90.27	72.44	154.93
KHO Based DeepNN	89.15	73.00	160.51	86.94	72.06	157.52	91.36	73.93	171.61
Modified AlexNet-SVM	90.94	74.31	177.04	89.69	73.50	182.06	92.19	75.12	179.72
I3DR-Net	93.23	76.94	159.13	94.25	76.41	174.45	92.21	77.48	150.02
JFOSTM	94.26	78.96	144.58	95.70	78.76	171.71	92.82	79.17	123.68
KHOSTM	95.25	81.85	127.82	97.05	81.93	144.93	93.45	81.78	117.03
SKO-DeepNN	95.59	84.51	82.56	97.13	84.79	95.07	94.05	84.22	74.02
FC2R + CHSTM	96.61	86.51	57.92	97.31	85.41	96.83	95.91	87.61	32.09

**TABLE 3:** Statistical analysis using the LIDC/IDRI dataset.

Methods	Accuracy			Sensitivity			Specificity		
	Best	Mean	Variance	Best	Mean	Variance	Best	Mean	Variance
SVM	74.05	60.17	106.36	77.41	60.49	126.09	70.70	59.84	91.88
KNN	81.70	63.99	147.32	78.20	62.07	130.98	85.20	65.92	165.76
ANN	82.75	64.80	142.00	79.23	63.29	117.02	86.27	66.32	170.36
CNN	84.38	66.67	150.73	82.33	65.74	143.80	86.42	67.60	161.69
DeepNN	86.22	69.01	144.26	84.54	68.49	149.74	87.90	69.54	145.06
BiLSTM	87.33	70.99	152.87	85.47	70.43	154.35	89.18	71.55	158.65
SFO Based DeepNN	88.35	71.93	154.76	86.44	71.41	160.90	90.27	72.44	154.93
KHO Based DeepNN	89.15	73.00	160.51	86.94	72.06	157.52	91.36	73.93	171.61
Modified AlexNet-SVM	90.94	74.31	177.04	89.69	73.50	182.06	92.19	75.12	179.72
I3DR-Net	93.23	76.94	159.13	94.25	76.41	174.45	92.21	77.48	150.02
JFOSTM	94.26	78.96	144.58	95.70	78.76	171.71	92.82	79.17	123.68
KHOSTM	95.25	81.85	127.82	97.05	81.93	144.93	93.45	81.78	117.03
SKO-DeepNN	95.59	84.51	82.56	97.13	84.79	95.07	94.05	84.22	74.02
FC2R + CHSTM	96.61	86.51	57.92	97.31	85.41	96.83	95.91	87.61	32.09

### 4.9 Computation time analysis

**FIGURE 12:**
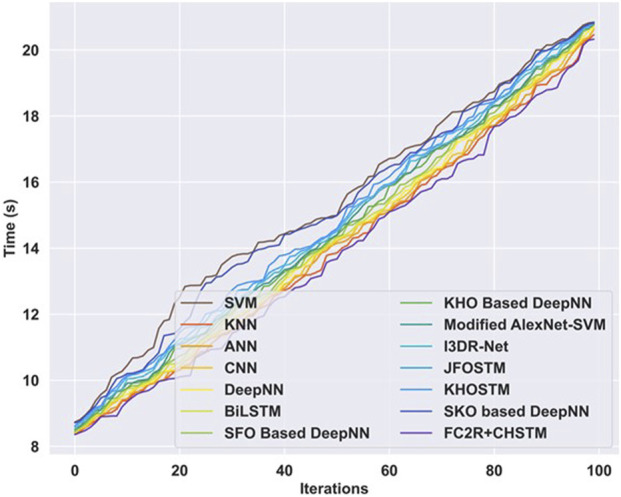
Computation time analysis.

Computation analysis is carried out between the proposed approach and the other existing techniques to analyze the computation efficiency. Figure 12 illustrates the results of the computation time analysis evaluated in terms of computation time with respect to the varying iterations. For iteration 100, the computation time attained by the proposed strategy is 20.33s, which is low compared to the other existing approaches. In the proposed approach, the CHO algorithm finetunes the hyperparameters of the CHSTM model and solves various high-dimensional challenging problems. Consequently, the CHO algorithm offers a fast convergence speed towards the optimal solution, enhances the training process, and reduces the computation time.

## 5 Conclusion

In conclusion, this research developed an innovative approach for lung nodule detection, combining deep learning and bio-inspired optimization techniques. The CHSTM model, powered by the CHO algorithm effectively detects lung nodules from CT images. The FC2R segmentation model combines the Resnet −101 deep learning model and the FCM technique that effectively improves the performance of the model. CHO is inspired by the food-searching and herding characteristics of cindaria and krill fish respectively. By adjusting model hyperparameters using the CHO algorithm, the accuracy of the model is improved. The statistical and texture features extract the pertinent features that improve nodule detection accuracy. The combination of the induced movement strategy of krill with the time control mechanism of the cnidaria improves the CHSTM model’s performance by attaining better detection accuracy and detecting lung nodules as malignant or benign. According to the findings of a performance comparison between the FC2R + CHSTM model and the existing approaches, the suggested model achieves 97.71% accuracy, 98.09% sensitivity, and 97.03% specificity for TP 80 using the LUNA-16 dataset. Further, the proposed approach obtained the accuracy, sensitivity, and specificity of 97.59%,96.77%, and 98.41% with k-fold validation utilizing the LIDC/IDRI dataset. Subsequent research endeavours to enhance the precision of the suggested approach by focusing network training on intricate instances.

## Data Availability

The original contributions presented in the study are included in the article/supplementary material, further inquiries can be directed to the corresponding author.
